# The Distribution of Phosphatidylcholine Species in Superficial-Type Pharyngeal Carcinoma

**DOI:** 10.1155/2017/5387913

**Published:** 2017-03-08

**Authors:** Seiji Ishikawa, Ichiro Tateya, Takahiro Hayasaka, Satoru Shinriki, Noritaka Masaki, Shigeru Hirano, Morimasa Kitamura, Manabu Muto, Shuko Morita, Mitsutoshi Setou, Juichi Ito

**Affiliations:** ^1^Ishikawa Clinic, 263 Iwakura Mikasa-cho, Sakyo-ku, Kyoto 606-0008, Japan; ^2^Department of Otolaryngology, Head and Neck Surgery, Graduate School of Medicine, Kyoto University, 54 Shogoin Kawahara-cho, Sakyo-ku, Kyoto 606-8507, Japan; ^3^Department of Cell Biology and Anatomy, Hamamatsu University School of Medicine, 1-20-1 Handayama, Higashi-ku, Hamamatsu 431-3192, Japan; ^4^Department of Otolaryngology, Head and Neck Surgery, Kyoto Prefectural University of Medicine, 465 Kajii-cho, Kamigyo-ku, Kyoto 602-8566, Japan; ^5^Department of Therapeutic Oncology, Graduate School of Medicine, Kyoto University, 54 Shogoin Kawahara-cho, Sakyo-ku, Kyoto 606-8507, Japan; ^6^Department of Gastroenterology and Hepatology, Kobe City Medical Center General Hospital, 2-2-1 Minatojimaminamimachi, Chuo-ku, Kobe City, Hyogo 650-0047, Japan; ^7^Shiga Medical Center Research Institute, 5-4-30, Moriyama, Moriyama, Shiga 524-8524, Japan

## Abstract

*Objectives*. Superficial-type pharyngeal squamous cell carcinoma (STPSCC) is defined as carcinoma in situ or microinvasive squamous cell carcinoma without invasion to the muscular layer. An exploration of the biological characteristics of STPSCC could uncover the invasion mechanism of this carcinoma. Phosphatidylcholine (PC) in combination with fatty acids is considered to play an important role in cell motility. Imaging mass spectrometry (IMS) is especially suitable for phospholipid analysis because this technique can distinguish even fatty acid compositions.* Study Design*. IMS analysis of frozen human specimens.* Methods*. IMS analysis was conducted to elucidate the distribution of PC species in STPSCC tissues. STPSCC tissue sections from five patients were analyzed, and we identified the signals that showed significant increases in the subepithelial invasive region relative to the superficial region.* Results*. Three kinds of PC species containing arachidonic acid, that is, PC (16:0/20:4), PC (18:1/20:4), and PC (18:0/20:4), were increased in the subepithelial invasive region.* Conclusion*. These results may be associated with the invasion mechanism of hypopharyngeal carcinoma.

## 1. Introduction

Pharyngeal squamous cell carcinoma (PSCC) is a life-threatening disease with aggressive features that include metastasis to cervical lymph nodes and distant organs and is associated with a high rate of local recurrence [1]. Because PSCC is difficult to detect in its early stages, this disease is frequently only diagnosed in an advanced stage, which worsens patient prognosis [2–4]. PSCC affects some of the most fundamental functions, including the ability to breathe and to eat. Therefore, early diagnosis of PSCC and a better understanding of the molecular mechanisms underlying the progression of this disease are urgently needed.

Superficial-type PSCC (STPSCC) is pathologically defined as carcinoma in situ or microinvasive squamous cell carcinoma without invading to the surrounding muscle layer [5]. Although STPSCC is considered to be a representative model of microinvasive carcinoma and thus could be useful for analyzing the mechanisms of PSCC cell invasion in its earliest stages [6], this condition is difficult to diagnose because lesions are rarely detected by standard endoscopy. The recently developed narrow band imaging (NBI) technique is an innovative optical technology that can clearly distinguish the microvascular structure of organ surfaces. While NBI has been routinely used to diagnose superficial esophageal carcinomas [7], NBI with magnifying endoscopy was also shown to be a powerful and promising tool for identifying STPSCC during routine endoscopic examinations [8–11]. In fact, development of this tool led to an increase in the number of patients diagnosed with STPSCC. This advance is particularly important because STPSCC can often be treated with minimally invasive endoscopic surgeries that may prevent progression to PSCC, which has a poorer prognosis because of local recurrence or the potential for neck lymph node metastasis. The anticipated increase in the number of STPSCC cases discovered by NBI will likely lead to a paradigm shift in treatment strategies for hypopharyngeal cancer. Together, these clinical improvements will enable more detailed pathological analyses of STPSCC, which could contribute to a better understanding of the molecular mechanisms that underlie PSCC invasion in its early stages.

Lipids are associated with cell membrane structure [12], cell proliferation [13], and inflammation [14]. Recent studies have focused on the relationship between cancer and lipids, especially fatty acid binding to lipids, in several types of cancer [15, 16]. Phosphatidylcholine (PC) is a lipid species that is the most abundant component of the cell membrane. Imaging mass spectrometry (IMS) based on matrix-assisted laser desorption/ionization (MALDI) time-of-flight (TOF) allows the two-dimensional visualization of the spatial distribution of molecules as relative signal intensities among the measured points on a tissue section. As such, MALDI-TOF is a very useful technique for analyzing PCs because, unlike other modalities, it can visualize even differences in fatty acids bound to PCs on tissue sections. Our previous studies for other cancers revealed that the amount of certain PCs as well as fatty acids was increased in cancerous regions [15–17].

In the present study, we compared mass spectra obtained between the superficial region and the subepithelial invasive region of STPSCC using IMS analysis to elucidate alterations in lipid distribution and composition during early invasion.

## 2. Materials and Methods

### 2.1. Ethics Statement

This study was performed in accordance with the Declaration of Helsinki and the guidelines for pathological specimen handling that were approved by the Institutional Review Board of Kyoto University and Hamamatsu University School of Medicine. We explained the nature and aims of this study to all subjects. All participants provided their written informed consent to participate in this study.

### 2.2. Sample Preparation

**Figure 1:**
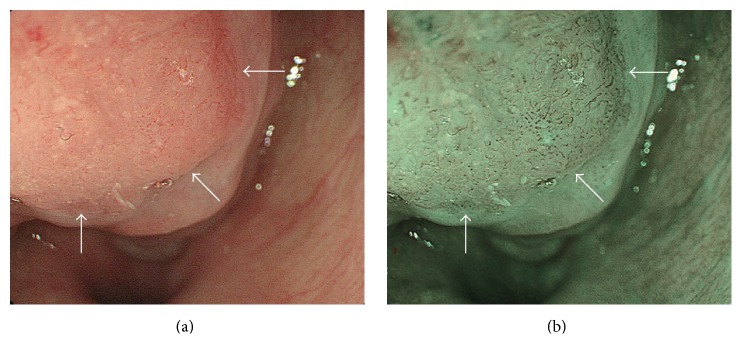
*Clinical endoscopic images of case 2 using conventional and NBI endoscopy*. (a) Squamous cell carcinoma of the hypopharynx. Conventional endoscopy shows slight reddish area in the postcricoid area (arrows). (b) Squamous cell carcinoma of the hypopharynx. NBI endoscopy shows a clearly demarcated brownish area in the postcricoid area (arrows).

Five samples from five Japanese patients included in the present study were obtained by endoscopic laryngeal pharyngeal surgery at Kyoto University Hospital. All the cases were detected by NBI with magnifying endoscopy as having regions of microvascular irregularities (Figure 1) and were then diagnosed as STPSCC with subepithelial invasion based on pathological examinations of formalin fixed and paraffin embedded tissue sections. All patients in this study were Japanese men between the ages of 53 and 76 y (mean age, 65.8 y). All patients experienced facial flushing following alcohol consumption and thus had an increased risk for pharyngeal cancer. Clinicopathological character of all cases was showed in Table 1. Tumor subsites were piriform sinus in 3 cases (case 3, 4, and 5), posterior wall in 1 (case 1), and postcricoid in 1 (case 2). There was no local recurrence in all cases after primary resection without adjuvant therapy. All tissue samples were placed in sterile tubes, immediately frozen in liquid nitrogen, and stored at −80°C until analysis. The tissue sections were sliced in 10 *μ*m thick sections using a cryostat (CM 1950; Leica, Wetzlar, Germany) and mounted onto indium-tin-oxide- (ITO-) coated glass slides.

### 2.3. Matrix Deposition

The matrix solution was prepared by dissolving 50 mg of 2,5-dihydroxybenzoic acid (DHB; Bruker Daltonics, Leipzig, Germany) in 1 mL 70% methanol and 20 mM potassium acetate. DHB is a widely used matrix for low-weight molecules. A thin matrix layer was applied to the surface of the tissue sections using a 0.2 mm nozzle airbrush (Procon Boy FWA Platinum; Mr. Hobby, Tokyo, Japan). The spraying distance was maintained at 15 cm from the tissue surface. The total amount of the matrix solution on each slide was 1 mL. The spraying technique enabled full matrix coverage over the entire tissue surface and facilitated cocrystallization of the matrix and biomolecules.

### 2.4. IMS Analysis

The tissue sections were analyzed using a matrix-assisted laser desorption/ionization-time-of-flight/time-of-flight (MALDI-TOF/TOF) type instrument, Ultraflex II TOF/TOF (Bruker Daltonics), which was equipped with a 355 nm smartbeam laser at 200 Hz repetition. Data were acquired in the positive-ion mode using an external calibration method with ions from DHB, angiotensin II, and bradykinin, which covered a mass-to-charge ratio (*m*/*z*) range of 100 to 1200. Calibration proteins were deposited on the surfaces of sample sections. Each raster scan was automatically performed in the regions of cancer and normal tissue. The interval between data points was 50 *μ*m, and 100 laser beam shots in a random walk mode for each data point were irradiated. The mass spectrometry parameters were manually optimized to obtain the highest sensitivity, with *m*/*z* values in the range of 500–900. All mass spectra were acquired automatically using FlexImaging 2.1 software (Bruker Daltonics).

### 2.5. Comparison of Signal Intensities between Superficial and Subepithelial Invasive Regions in STPSCC

Hematoxylin and eosin (HE) staining was performed on consecutive sections to distinguish the superficial and subepithelial invasive regions. To compare mass spectra, the regions of interest (ROIs) in the subepithelial invasive and superficial epithelial region were defined based on the HE staining results using SIMtools software (in-house software; Shimadzu Corporation). The top 50 peaks (excluding isotopic peaks) were identified from the defined ROIs, and the statistical difference was determined by Welch's *t*-test. Differences with *P* < 0.01 were considered significant. IMS and statistical analyses were performed on all cases in the same manner, and the *m*/*z* values that reproducibly appeared in all cases were chosen. These signal characteristics in the subepithelial region were employed for MS/MS analysis to identify the relevant molecules.

### 2.6. MS/MS Analysis

MS/MS analysis was performed on tissue sections in the positive-ion mode using a QSTAR Elite (Applied Biosystems/MDS Sciex, Foster City, CA, USA), which is a hybrid quadrupole/TOF mass spectrometer equipped with an orthogonal MALDI source and a pulsed Nd:YAG laser. The Metabolite MS Search Database (http://www.hmdb.ca/spectra/spectra/ms/search) was referenced to determine the molecular species of phospholipids.

## 3. Results

### 3.1. Distinct Molecular Distribution between Superficial and Subepithelial Invasive Regions in STPSCC

**Figure 2:**
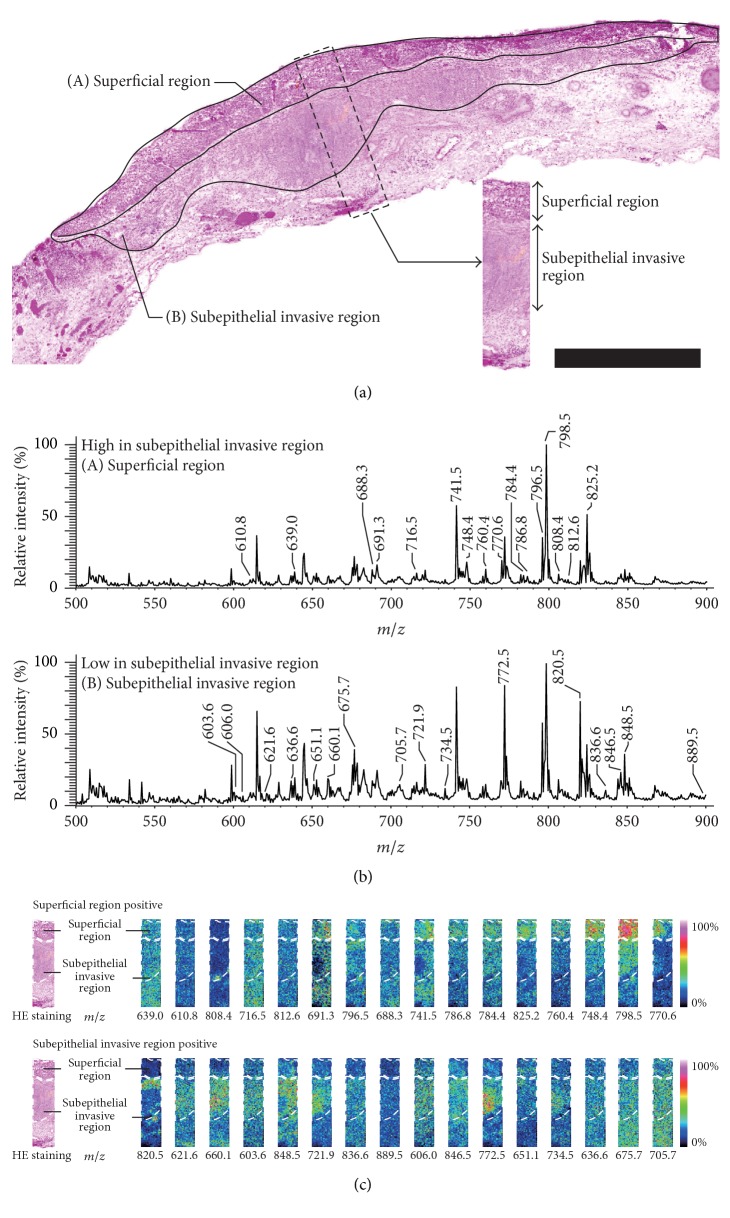
*IMS analyses of case 1*. (a) HE staining was performed to distinguish the ROIs on the superficial and subepithelial invasive regions in STPSCC delimited by the line (scale bar: 1.0 mm). The box with the dashed line indicates the representative region of STPSCC. (b) Mass spectra of the superficial (A) and subepithelial invasive (B) regions of STPSCC in case 1 are shown. (c) Images of ions shown in Table 2 were reconstructed on the representative region. Upper and lower columns show the ion images of the signals with higher and lower intensity in the subepithelial invasive region and superficial region, respectively.

To elucidate the distribution of PCs in STPSCC tissues, we performed IMS analyses and compared the mass spectra according to the HE staining results (Figure 2(a)) for the ROIs of the superficial region and subepithelial invasive region.  Figure 2(b) shows the average mass spectra obtained from ROIs in the superficial (A) and subepithelial invasive (B) regions of STPSCC in the representative case shown in Figure 2(a). The horizontal and vertical axes indicate *m*/*z* and relative intensity, respectively. The highest intensity of *m*/*z* 798.5 is shown as 100 and is referred to as the base peak.

For a systematic comparison, 50 peaks were identified according to their intensities and statistically assessed by performing Welch's *t*-test between the superficial region and subepithelial invasive region. The average intensities (±standard error) for each peak in the superficial regions and subepithelial invasive regions were calculated around the measured points. In the superficial region and subepithelial invasive region ROIs there were 586 and 708 points in case 1, 561 and 502 points in case 2, 206 and 533 points in case 3, 296 and 991 points in case 4, and 149 and 350 points in case 5.

As a result, the numbers of *m*/*z* values that appeared to be significantly higher in the subepithelial invasive region compared to the superficial region (*P* < 0.01) were 32 for case 1 (as shown in Figure 2(b)), 25 in case 2, 17 in case 3, 25 in case 4 and 22 in case 5 (Tables 2–6). In Tables 2–6, the *m*/*z* values are sorted in descending order of the fold change that was calculated by dividing the signal intensity in the subepithelial invasive region by the signal intensity in the superficial region. Isotopic peaks and those peaks that lacked significant differences were excluded. All *m*/*z* values in Table 2 were assigned to the mass spectra shown in Figure 2(b), while Figure 2(c) shows the ion image of the representative region delimited by the dashed line in Figure 2(a). The pseudocolor range in each ion image was optimized manually to show clear distributions.

### 3.2. Identification of Signals That Showed Increases for All Cases

**Figure 3:**
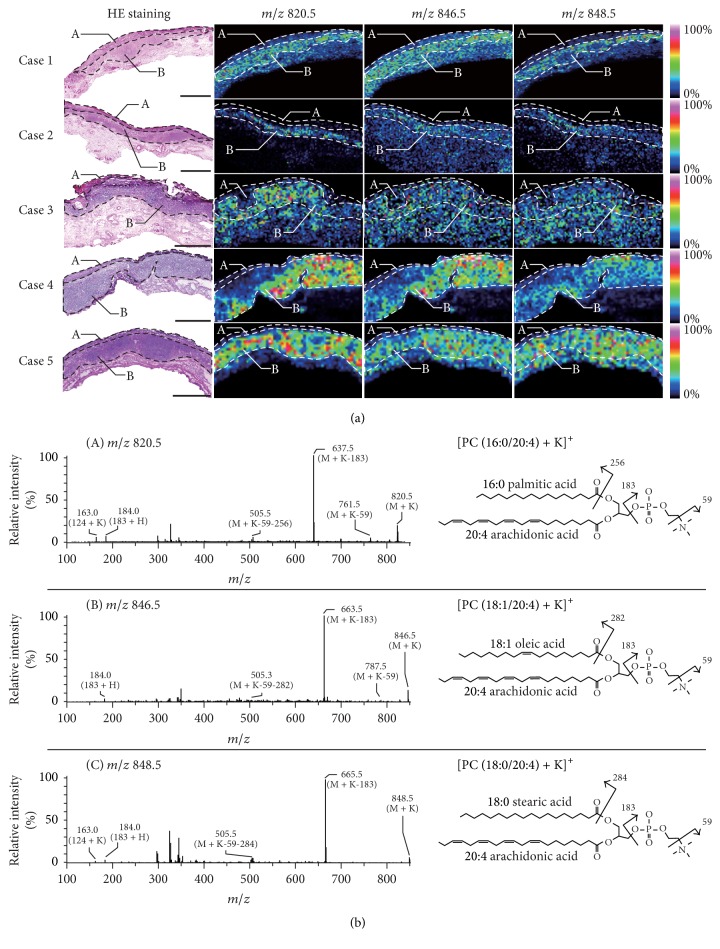
*Ion images of signals in the subepithelial invasive region that were increased in all STPSCC cases and molecular identification*. (a) Ion images of signals in the subepithelial invasive region that were increased in all STPSCC cases. The white dashed lines indicate ROIs of the superficial (A) and subepithelial invasive (B) regions in STPSCC (scale bar: 1.0 mm). (b) (A) Results of MS/MS analysis of *m*/*z* 820.5. The product ions from *m*/*z* 820.5 as a precursor ion were obtained in the subepithelial invasive region of STPSCC. This biomolecule was identified as [PC (16:0/20:4) + K]^+^. (B) The precursor peak at *m*/*z* 846.5 was identified in the same manner as [PC (18:1/20:4) + K]^+^. (C) The precursor peak at *m*/*z* 848.5 was identified as [PC (18:0/20:4) + K]^+^. All identified molecules that had a higher signal intensity in the subepithelial invasive region of STPSCC contained an arachidonic acid (20:4).

To explore potential therapeutic targets for STPSCC, we focused on signals that were significantly increased in the subepithelial invasive region compared to the superficial region (Table 7). Three *m*/*z* values at *m*/*z* 820.5, *m*/*z* 846.5, and *m*/*z* 848.5 were increased for all cases (Table 7). We confirmed that these three signals were specifically increased in the subepithelial invasive region (B) relative to the superficial region (A) in the ion images (Figure 3(a)).

### 3.3. Molecular Identification

The three *m*/*z* signals that were increased in the subepithelial invasive regions of all cases were subjected to MS/MS analysis to identify the corresponding molecular structures (Figure 3(b)). The Metabolite MS Search Database (http://www.hmdb.ca/spectra/spectra/ms/search) was used as a reference. From the MS/MS spectrum shown in Figure 3(b), the peak at *m*/*z* 820.5 was identified as phosphatidylcholine (PC) due to the neutral losses of 59 Da (*m*/*z* 761.5) and 183 Da (*m*/*z* 637.5) [18, 19]. As potassium salt was added to the matrix solution to simplify the adduct ion [14] in the present study the peak at *m*/*z* 820.5 could be identified as [PC (36:4) + K]^+^ according to a Metabolite MS Search. Moreover, a minor peak detected at *m*/*z* 505.5 was consistent with the neutral loss of (59 + 256 Da) from the precursor peak. The molecular weight of 256 Da corresponds to the fatty acid 16:0 so that the peak at *m*/*z* 820.5 was identified as [PC (16:0/20:4) + K]^+^. In the same manner, we concluded that *m*/*z* 846.5 and *m*/*z* 848.5 corresponded to [PC (18:1/20:4) + K]^+^ and [PC (18:0/20:4) + K]^+^. Importantly, these three identified PCs had arachidonic acid (20:4) in common.

## 4. Discussion

The results of this study showed that the fatty acids bound to PCs in the subepithelial invasive region of STPSCC differed from those that bound to PCs in the superficial region. PC is the most abundant component of the cell membrane, and membrane fluidity is thought to depend strictly on the fatty acid species that bind to PCs [12, 20]. These interactions could therefore affect cell mobility in cancer invasion and metastasis. The changes in PC/fatty acid interactions observed in STPSCC in this study may have occurred for several reasons. First, given that fatty acids can influence both cell shape and membrane fluidity, the cell membrane in STPSCC may have undergone autonomous alterations when the cancer invaded into the subepithelial region [12]. Furthermore, changes in cancer cell membrane fluidity may influence the biological behaviors of cancers, including invasion and metastasis. Unsaturated fatty acids such as AA are known to be contained in cell membrane phospholipids and can lead to greater variations in cell membrane fluidity. Our results indicated that increased amounts of AA-containing phospholipids in the cell membrane may promote subepithelial invasion of STPSCC. Second, fatty acid binding to PC in the subepithelial invasive region may be altered by cell interactions and inflammation associated with the invasion of STPSCC cancer cells into subepithelial tissues. Increased levels of PC binding to AA could also arise from the cell membranes of lymphocytes. Lymphocyte accumulation is known to be associated with cancer invasion [14], and following the initial appearance of lymphocytes oncogenic changes may induce an inflammatory microenvironment that promotes tumor development [21]. In our results, many lymphocytes were observed in the subepithelial invasive region of STPSCC (Figure 2(a)). Hanada et al. reported that PCs with AA in the spinal cord were elevated in inured spinal cord tissue. These authors hypothesized that the elevation of PC with AA may be derived from invasive immune cells and there may be a molecular flux through the AA cascade in immune cells that results in increased AA storage in the form of AA binding to cell membrane PCs [22]. Their findings suggest that the increased level of PC seen here in the subepithelial invasive region of STPSCC could reflect an increase in cell membrane components induced by an inflammatory microenvironment that may be present in the subepithelial invasive region of STPSCC.

Chronic inflammation and stimulation were also reported to contribute to cancer development [14, 23–25]. Indeed, a chronic increase in inflammatory mediators in head and neck squamous cell carcinoma (HNSCC) can lead to increased tumor promotion, invasion, angiogenesis, and metastasis [26]. AA is involved in cellular signaling as a lipid second messenger in the regulation of signaling pathways. Phospholipase A2 (PLA2) activity releases fatty acids such as AA from phospholipids and promotes tumor progression by providing extracellular regulation of the tumor microenvironment to trigger cell migration and invasion. Meanwhile, cyclooxygenase-2 (COX-2) converts arachidonic acid (AA) to prostaglandins (PGs) that in turn induce inflammatory reactions in damaged tissue. Aspirin acting as a COX-inhibitor can reduce the production of PGs and other inflammatory mediators. Multiple meta-analyses and reviews have concluded that daily use of aspirin reduces the long-term risk of death due to gastrointestinal cancer as well as head and neck squamous cell carcinoma [27, 28]. Thus, functional analyses that focus on metabolic enzymes such as PLA2 could support the validity of the results of this study.

## 5. Conclusions

The present study is the first to reveal changes in PCs associated with STPSCC infiltration to subepithelial tissue and revealed that three PCs with arachidonic acids, that is, PC (16:0/20:4), PC (18:1/20:4), and PC (18:0/20:4), were increased in the subepithelial invasive region relative to the superficial region of hypopharyngeal carcinoma. Our results could thus contribute to a better understanding of the mechanisms involved in hypopharyngeal carcinoma invasion.

## Figures and Tables

**Table 1:** Clinicopathological character of all cases.

	Tumor subsite	Age	Sex	Pathological tumor diameter
Case 1	Posterior wall	53	Male	10 mm
Case 2	Postcricoid	75	Male	19 mm
Case 3	Piriform sinus	76	Male	12 mm
Case 4	Piriform sinus	64	Male	6 mm
Case 5	Piriform sinus	61	Male	14 mm

**Table 2:** *m*/*z* values showing a significant difference between the superficial region and subepithelial invasion region in STPSCC (case  1).

*m*/*z*	Superficial region	Subepithelial invasive region	Fold change	*P* value
820.5	24.1 ± 1.2	40.7 ± 1.3	1.69	4.2*E* − 21
621.6	3.3 ± 0.1	5.3 ± 0.1	1.61	6.8*E* − 39
660.1	7.4 ± 0.2	11.4 ± 0.2	1.54	1.8*E* − 42
603.6	2.3 ± 0.1	3.4 ± 0.1	1.48	1.1*E* − 28
848.5	14.0 ± 0.4	19.8 ± 0.5	1.41	4.6*E* − 20
721.9	13.1 ± 0.3	17.1 ± 0.3	1.31	1.7*E* − 22
836.6	5.1 ± 0.1	6.5 ± 0.1	1.27	6.7*E* − 14
889.5	3.2 ± 0.1	4.0 ± 0.1	1.25	2.6*E* − 12
606.0	3.7 ± 0.1	4.5 ± 0.1	1.22	1.3*E* − 10
846.5	10.6 ± 0.3	12.8 ± 0.3	1.21	5.4*E* − 08
772.5	38.8 ± 1.0	46.8 ± 1.2	1.21	3.9*E* − 07
651.1	8.4 ± 0.2	9.6 ± 0.2	1.14	7.2*E* − 06
734.5	6.1 ± 0.1	6.9 ± 0.2	1.13	5.7*E* − 04
636.6	9.0 ± 0.2	10.0 ± 0.2	1.11	4.3*E* − 06
675.7	14.6 ± 0.3	16.2 ± 0.2	1.11	3.7*E* − 06
705.7	7.9 ± 0.1	8.6 ± 0.1	1.09	1.1*E* − 04
610.8	5.9 ± 0.1	5.5 ± 0.1	0.93	4.5*E* − 03
639.0	11.8 ± 0.2	11.0 ± 0.2	0.93	6.4*E* − 03
808.5	6.4 ± 0.2	5.7 ± 0.1	0.89	6.2*E* − 04
716.5	10.6 ± 0.2	9.4 ± 0.2	0.89	7.5*E* − 07
812.6	6.3 ± 0.1	5.4 ± 0.1	0.86	3.4*E* − 07
691.3	17.1 ± 0.4	14.0 ± 0.3	0.82	5.1*E* − 11
796.5	40.2 ± 1.1	32.1 ± 0.8	0.80	1.3*E* − 09
688.3	13.4 ± 0.3	10.0 ± 0.2	0.75	1.2*E* − 19
741.5	60.8 ± 1.6	45.3 ± 1.0	0.75	3.4*E* − 16
786.5	8.5 ± 0.2	6.2 ± 0.1	0.73	9.5*E* − 18
784.5	9.6 ± 0.2	6.9 ± 0.1	0.72	2.3*E* − 21
825.2	9.8 ± 0.3	6.0 ± 0.1	0.61	3.9*E* − 37
760.4	14.1 ± 0.4	8.6 ± 0.2	0.61	9.4*E* − 31
748.5	19.2 ± 0.5	10.8 ± 0.2	0.56	8.7*E* − 54
798.5	106.7 ± 2.7	55.8 ± 1.3	0.52	4.1*E* − 57
770.5	21.3 ± 0.7	9.6 ± 0.2	0.45	5.1*E* − 49

**Table 3:** *m*/*z* values showing a significant difference between the superficial region and subepithelial invasion region in STPSCC from case  2.

*m*/*z*	Superficial region	Subepithelial invasive region	Fold change	*P* value
820.5	22.1 ± 1.5	69.4 ± 2.8	3.13	4.7*E* − 45
782.5	9.5 ± 0.4	14.1 ± 0.6	1.48	1.1*E* − 11
848.5	8.8 ± 0.4	12.6 ± 0.4	1.44	7.0*E* − 11
637.2	9.1 ± 0.3	12.1 ± 0.3	1.33	8.4*E* − 13
772.5	46.5 ± 1.9	60.6 ± 2.1	1.30	8.8*E* − 07
628.9	10.6 ± 0.3	13.7 ± 0.3	1.28	3.7*E* − 11
790.8	6.5 ± 0.2	8.3 ± 0.3	1.27	1.5*E* − 07
846.5	9.4 ± 0.3	11.7 ± 0.4	1.25	6.0*E* − 06
844.5	9.0 ± 0.3	10.7 ± 0.3	1.19	1.2*E* − 04
734.6	7.5 ± 0.3	8.9 ± 0.3	1.18	1.8*E* − 03
876.6	3.7 ± 0.1	4.3 ± 0.2	1.15	6.1*E* − 03
716.4	11.5 ± 0.4	10.1 ± 0.3	0.88	7.0*E* − 03
676.8	16.5 ± 0.5	14.3 ± 0.4	0.87	3.1*E* − 04
644.9	38.0 ± 0.8	32.4 ± 0.7	0.85	1.7*E* − 07
808.5	7.1 ± 0.3	6.0 ± 0.2	0.85	1.8*E* − 03
659.7	13.2 ± 0.6	11.1 ± 0.4	0.84	3.4*E* − 03
615.1	64.3 ± 1.5	53.5 ± 1.3	0.83	1.1*E* − 07
714.2	10.5 ± 0.3	7.6 ± 0.2	0.72	3.3*E* − 12
760.5	15.9 ± 0.6	10.7 ± 0.4	0.67	8.6*E* − 12
796.5	18.8 ± 0.8	12.2 ± 0.5	0.65	5.5*E* − 12
688.4	29.8 ± 1.1	18.0 ± 0.6	0.60	6.1*E* − 20
798.5	150.6 ± 5.8	87.3 ± 2.9	0.58	2.1*E* − 21
741.5	70.7 ± 2.9	38.5 ± 1.2	0.54	4.3*E* − 23
770.5	23.5 ± 1.0	11.1 ± 0.4	0.47	9.8*E* − 27
824.5	32.6 ± 1.3	14.9 ± 0.5	0.46	4.0*E* − 34

**Table 4:** *m*/*z* values showing a significant difference between the superficial region and subepithelial invasion region in STPSCC from case  3.

*m*/*z*	Superficial region	Subepithelial invasive region	Fold change	*P* value
820.5	1.0 ± 0.2	4.5 ± 0.4	4.54	1.7*E* − 13
770.1	0.2 ± 0.0	0.7 ± 0.1	4.24	3.2*E* − 07
848.5	0.4 ± 0.1	1.2 ± 0.1	3.26	1.7*E* − 09
846.5	0.2 ± 0.0	0.4 ± 0.0	2.58	2.7*E* − 06
844.5	0.4 ± 0.1	0.8 ± 0.1	2.27	7.7*E* − 05
822.5	2.3 ± 0.4	4.9 ± 0.4	2.17	7.9*E* − 07
772.5	9.3 ± 1.3	16.4 ± 1.1	1.77	4.0*E* − 05
746.1	0.5 ± 0.1	0.9 ± 0.1	1.65	8.4*E* − 03
691.1	1.4 ± 0.2	0.7 ± 0.1	0.52	6.0*E* − 03
638.9	2.0 ± 0.4	0.5 ± 0.1	0.26	9.4*E* − 05
688.4	3.9 ± 0.6	0.9 ± 0.1	0.23	1.4*E* − 06
615.0	9.1 ± 1.9	1.7 ± 0.2	0.19	2.3*E* − 04
676.8	4.4 ± 1.3	0.5 ± 0.1	0.10	3.4*E* − 03
680.8	1.9 ± 0.6	0.2 ± 0.0	0.09	6.7*E* − 03
808.2	2.3 ± 0.4	0.2 ± 0.0	0.09	6.5*E* − 06
655.1	3.8 ± 0.6	0.2 ± 0.0	0.06	6.0*E* − 08
616.2	15.7 ± 2.0	0.8 ± 0.1	0.05	8.6*E* − 12

**Table 5:** *m*/*z* values showing a significant difference between the superficial region and subepithelial invasion region in STPSCC from case  4.

*m*/*z*	Superficial region	Subepithelial invasive region	Fold change	*P* value
770.0	13.2 ± 1.3	42.1 ± 0.9	3.20	4.5*E* − 56
821.5	37.6 ± 3.1	112.4 ± 2.1	2.99	1.9*E* − 64
820.5	103.4 ± 7.6	296.5 ± 5.2	2.87	6.3*E* − 70
804.5	4.1 ± 0.3	10.1 ± 0.3	2.46	4.4*E* − 39
844.4	10.7 ± 0.8	25.6 ± 0.5	2.39	1.8*E* − 40
846.5	14.8 ± 1.1	33.3 ± 0.6	2.25	6.4*E* − 38
616.2	2.6 ± 0.2	4.7 ± 0.3	1.83	2.5*E* − 07
655.1	1.1 ± 0.1	1.9 ± 0.1	1.74	4.4*E* − 06
848.5	17.8 ± 1.1	30.9 ± 0.6	1.73	7.4*E* − 21
808.1	1.4 ± 0.1	2.3 ± 0.2	1.69	1.3*E* − 05
822.5	50.3 ± 2.0	77.6 ± 1.4	1.54	7.4*E* − 25
782.5	16.3 ± 0.7	22.5 ± 0.5	1.38	3.0*E* − 13
796.5	99.4 ± 3.6	133.1 ± 2.3	1.34	7.1*E* − 14
746.0	11.0 ± 0.5	14.2 ± 0.3	1.29	2.2*E* − 07
677.8	9.3 ± 0.3	11.1 ± 0.2	1.20	7.2*E* − 06
676.8	8.5 ± 0.3	10.0 ± 0.2	1.18	2.2*E* − 05
675.8	8.6 ± 0.3	10.0 ± 0.2	1.16	1.4*E* − 04
722.0	7.4 ± 0.3	8.4 ± 0.2	1.15	4.8*E* − 03
772.5	64.1 ± 2.4	73.0 ± 1.3	1.14	1.6*E* − 03
748.0	16.7 ± 0.7	14.6 ± 0.3	0.88	6.4*E* − 03
824.5	36.0 ± 1.5	31.2 ± 0.7	0.87	4.8*E* − 03
688.4	23.7 ± 0.8	15.6 ± 0.2	0.66	2.1*E* − 18
691.1	10.2 ± 0.4	5.1 ± 0.1	0.50	1.0*E* − 29
741.5	60.0 ± 2.1	29.5 ± 0.6	0.49	2.1*E* − 34
770.7	13.7 ± 1.0	3.6 ± 0.1	0.26	8.5*E* − 21

**Table 6:** *m*/*z* values showing a significant difference between the superficial region and subepithelial invasion region in STPSCC from case  5.

*m*/*z*	Superficial region	Subepithelial invasive region	Fold change	*P* value
769.9	1.2 ± 0.2	9.4 ± 0.7	7.89	3.4*E* − 28
820.5	16.8 ± 1.5	62.6 ± 3.7	3.73	4.1*E* − 26
844.5	4.3 ± 0.5	14.8 ± 0.7	3.43	3.5*E* − 28
848.5	5.5 ± 0.6	11.3 ± 0.6	2.06	5.6*E* − 12
846.5	4.3 ± 0.5	7.7 ± 0.5	1.79	7.3*E* − 07
721.9	7.9 ± 1.0	5.2 ± 0.4	0.66	1.3*E* − 02
822.4	32.7 ± 3.5	17.6 ± 0.9	0.54	1.0*E* − 04
745.9	5.3 ± 0.7	2.7 ± 0.2	0.52	1.0*E* − 03
772.5	83.8 ± 7.0	41.5 ± 1.9	0.50	1.4*E* − 07
614.9	2.7 ± 0.3	1.3 ± 0.1	0.49	9.4*E* − 05
796.5	47.1 ± 5.0	22.8 ± 1.3	0.48	1.5*E* − 05
808.5	0.8 ± 0.1	0.4 ± 0.1	0.46	1.7*E* − 03
824.5	49.8 ± 4.6	12.8 ± 0.6	0.26	5.2*E* − 12
748.0	22.1 ± 2.5	5.5 ± 0.3	0.25	2.6*E* − 09
770.5	3.6 ± 0.5	0.9 ± 0.1	0.24	1.3*E* − 06
798.5	120.2 ± 12.0	28.9 ± 1.5	0.24	5.9*E* − 11
688.4	10.1 ± 1.2	2.4 ± 0.2	0.24	1.0*E* − 08
826.5	19.2 ± 2.1	4.1 ± 0.2	0.22	3.0*E* − 10
691.1	9.2 ± 1.3	1.8 ± 0.1	0.19	2.2*E* − 07
782.5	20.3 ± 1.9	3.9 ± 0.3	0.19	3.6*E* − 13
741.5	45.8 ± 4.9	8.7 ± 0.6	0.19	8.3*E* − 11
758.5	7.1 ± 1.0	1.0 ± 0.1	0.14	2.8*E* − 08

**Table 7:** *m*/*z* values that were commonly increased in the subepithelial invasive region.

Case 1	Case 2	Case 3	Case 4	Case 5
820.5^*∗*^	820.5^*∗*^	820.5^*∗*^	770.0	769.9
621.6	782.5	770.1	821.5	820.5^*∗*^
660.1	848.5^*∗*^	848.5^*∗*^	820.5^*∗*^	844.5
603.6	637.2	846.5^*∗*^	804.5	848.5^*∗*^
848.5^*∗*^	772.5	844.5	844.4	846.5^*∗*^
721.9	628.9	822.5	846.5^*∗*^	
836.6	790.8	772.5	616.2	
889.5	846.5^*∗*^	746.1	655.1	
606.0	844.5		848.5^*∗*^	
846.5^*∗*^	734.6		808.1	
772.5	876.6		822.5	
651.1			782.5	
734.5			796.5	
636.6			746.0	
675.7			677.8	
705.7			676.8	
			675.8	
			722.0	
			772.5	

^*∗*^ *m*/*z* values (*m*/*z* 820.5, *m*/*z* 846.5, and *m*/*z* 848.5) that showed higher intensities in the subepithelial invasive region relative to the superficial region for all cases.
